# Tea and Coffee

**Published:** 1890-08

**Authors:** 


### TEA AND COFFEE.

Abstract of a Lecture by J. H. Kellogg, M. D., of Battle Creek Sanitarium.

Tea and coffee rank among the worst narcotics used by civilized peoples. Alcohol may be placed at the head of the list, tobacco next, tea and coffee next, and then opium. I do not mean to say that in an individual instance, the use of tea and coffee is usually to be regarded as worse than the use of opium, but that the aggregate of evil arising from the use of these narcotics is very greatly on the side of tea and coffee.

Both of them contain an alkaloid known as theine or caffeine. The substances are indentical in each and the two names arise from the fact that two different chemists were making analyses about the same time, the one on tea and the other on coffee and each gave a name to his own discovery. It is a curious coincidence that the same substance should be found in the seed of the coffee tree and in the leaves of the tea plant, the one growing in Arabia and the other in China.

Careful investigations show that theine is a deadly poison when administered to animals. Seven and a half grains will kill a cat in a few moments. Twelve grains have been known to produce serious results in human beings; twenty grains would probably be almost or quite fatal. It is not so fatal in small doses as arsenic or strychnine and most people are habituated to its use in much the same way that the opium eater takes his deadly drug with safety.

An incident in connection with the British soldiery in Africa illustrates the deadly influence of tea upon animals. The soldiers carried their corn in sacks and their tea in sacks. One time some corn was put into a tea sack which was supposed to be empty but which contained quite a quantity of finely pulverized tea. In feeding the corn to the horses the portion in the bottom of the sack was given to a fine horse which, being very hungry, ate it down tea and all. In a short time the horse began to show symptoms of violent poisoning; it plunged and reared about and finally fell over the brink of a precipice and lay there writhing in great agony until an officer thrust a dagger through its heart to end its misery.

The most common effect of tea is to keep people awake. A person who is exhausted and nervous and his whole body needing rest, takes a cup of tea and he not only finds the feeling of fatigue gone but that he cannot go to sleep. A drug which will keep a person awake under such circumstances must be very powerful.

Theine is a near relative of cocaine, an alkaloid with powerful anesthetic properties. A little cocaine put into a throat which is red and inflamed will blanch it so as to appear absolutely bloodless, so great is its effect in producing contraction of the blood vessels. Theine taken into the stomach produces a similar effect, only not quite so bad. It causes contraction of the blood vessels of the stomach, diminishes the secretions, paralyzes the nerves and lessens the activities of the stomach. The gastric juice is not poured out in so large quantities and is less potent in its offices. So with the manufacture of peptones. The contraction of the blood vessels also prevents proper absorption.

Tannin, the other chief active principle of tea, is used medicinally as an astringent, and its effect upon the stomach is not unlike that of theine, only less powerful.

Experience shows that the effects of tea and coffee are to impair digestion. Experiments have been made thousands of times not only in chemical laboratories, but in homes, with the same result. These things are of no nutritive value whatever beyond the little milk and sugar taken with them, and are positively pernicious, making thousands and thousands of dyspeptics yearly. Catarrh of the stomach is produced in many instances from the use of tea. The constriction of the blood vessels mentioned is always followed by relaxation and then the amount of secretion poured out is too great, and if a catarrh of the stomach becomes established, it is one of the most difficult of diseases to cure. Physicians who give attention to the stomach are beginning to realize that tea dyspepsia is a very common malady.

A few figures will show how much of this poison the tea drinker is taking in. Strong tea contains six per cent. of theine, which is about three and three-fifths grains to a drachm. A teaspoonful of tea is a drachm, and hence according to the old rule of a teaspoonful to the cup, there would be three and three-fifths grains of theine in a single cup of strong tea. Seven grains will kill a cat, so it would only take two and a half cups to make a fatal dose for a cat.

Dr. Edward Smith of England, made a decoction of four ounces of coffee which he and his assistant drank together. In fifteen minutes they fell upon the floor unconscious and remained so for thirty minutes. Probably if either had had constitutional weakness of the heart, the dose would have been fatal.

Temperance people are making a great mistake in establishing tea and coffee houses as a substitute for the saloon, for it is really substituting evil for evil. It is doubtless true that a great deal of social conspiracy is hatched over tea and coffee cups, and society as well as digestion, would be better off if the custom was discontinued. Hot milk is nourishing, refreshing and palatable. It has every advantage of tea and coffee, with no drawbacks whatever as a wholesome temperance drink.

